# Morphological Plasticity in a Sulfur-Oxidizing Marine Bacterium from the SUP05 Clade Enhances Dark Carbon Fixation

**DOI:** 10.1128/mBio.00216-19

**Published:** 2019-05-07

**Authors:** Vega Shah, Xiaowei Zhao, Rachel A. Lundeen, Anitra E. Ingalls, Daniela Nicastro, Robert M. Morris

**Affiliations:** a School of Oceanography, University of Washington, Seattle, Washington, USA; b University of Texas Southwestern Medical Center, Dallas, Texas, USA; Oregon State University

**Keywords:** OMZ, SUP05, chemoautotrophy, oxygen, sulfur

## Abstract

Sulfur-oxidizing bacteria from the SUP05 clade are abundant in anoxic and oxygenated marine waters that appear to lack reduced sources of sulfur for cell growth. This raises questions about how these chemosynthetic bacteria survive across oxygen and sulfur gradients and how their mode of survival impacts the environment. Here, we use growth experiments, proteomics, and cryo-electron tomography to show that a SUP05 isolate, “*Candidatus* Thioglobus autotrophicus,” is amorphous in shape and several times larger and stores considerably more intracellular sulfur when it respires oxygen. We also show that these cells can use diverse sources of reduced organic and inorganic sulfur at submicromolar concentrations. Enhanced cell size, carbon content, and metabolic activity of the aerobic phenotype are likely facilitated by a stabilizing surface-layer (S-layer) and an uncharacterized form of FtsZ-less cell division that supports morphological plasticity. The additional sulfur storage provides an energy source that allows cells to continue metabolic activity when exogenous sulfur sources are not available. This metabolic flexibility leads to the production of more organic carbon in the ocean than is estimated based solely on their anaerobic phenotype.

## Abstract (precis)

Identifying shifts in microbial metabolism across redox gradients will improve efforts to model marine oxygen minimum zone (OMZ) ecosystems. Here, we show that aerobic morphology and metabolism increase cell size, sulfur storage capacity, and carbon fixation rates in “*Ca*. Thioglobus autotrophicus,” a chemosynthetic bacterium from the SUP05 clade that crosses oxic-anoxic boundaries.

## INTRODUCTION

Sulfide is a primary energy source for chemolithotrophic bacteria in anoxic ecosystems. In the oceans, sulfide supports the growth of planktonic sulfur-oxidizing bacteria in marine oxygen minimum zones (OMZs), fjords and enclosed seas with anoxic bottom waters, and in deep-sea hydrothermal systems where anoxic fluids enriched in sulfide mix with seawater (1). Evidence that sulfur bacteria are abundant and active in nonsulfidic waters, where sulfide is below detection (<1 μM S^2−^), led to the suggestion that a cryptic sulfur cycle in marine OMZs influences biogeochemical cycles in the global ocean (2).

Sulfur-oxidizing bacteria from the SUP05 clade often dominate microbial communities in marine OMZs, fjords, enclosed seas, and deep-sea hydrothermal systems (3–9). In anoxic waters, their populations have critical roles in mediating dark carbon fixation (chemoautotrophy), denitrification (nitrogen loss), and sulfur oxidation (10–14). For example, field estimates of SUP05 carbon fixation in anoxic waters suggest that SUP05 cells fix between 1.3 and 592 nmol C liter^−1 ^day^−1^ (10), or up to 10% of surface primary production (11). Additionally, several studies have now indicated that chemolithotrophic members of the SUP05 clade both have the genetic potential to respire oxygen and are active in oxic environments (5, 10, 15–18). Despite the importance of SUP05 metabolism to dark carbon fixation and denitrification in the ocean, neither the biological underpinnings of this apparent metabolic flexibility nor the sources of reduced sulfur that are required to fuel their activity in oxygenated waters are currently understood.

We used a cultured representative isolated from a seasonally anoxic fjord, “*Candidatus* Thioglobus autotrophicus,” to test the hypothesis that phenotypic plasticity allows this representative member of the SUP05 clade to inhabit both oxic and anoxic waters with important implications for biogeochemical cycles. “*Ca*. Thioglobus autotrophicus” is a facultative anaerobic chemolithoautotroph that requires a reduced source of sulfur and uses nitrate or oxygen as a terminal electron acceptor (19). We used growth experiments, cryo-electron tomography (cryo-ET), and label-free quantitative proteomics to show that the growth rate, morphology, and metabolism of “*Ca*. Thioglobus autotrophicus” are different under oxic versus anoxic conditions and with different sulfur availability. Our findings illustrate the significance of phenotypic plasticity not only for SUP05 survival across redox gradients but also for marine biogeochemical cycles of C, N, and S.

## RESULTS AND DISCUSSION

### Growth on reduced sources of sulfur and dissolved oxygen.

**FIG 1:**
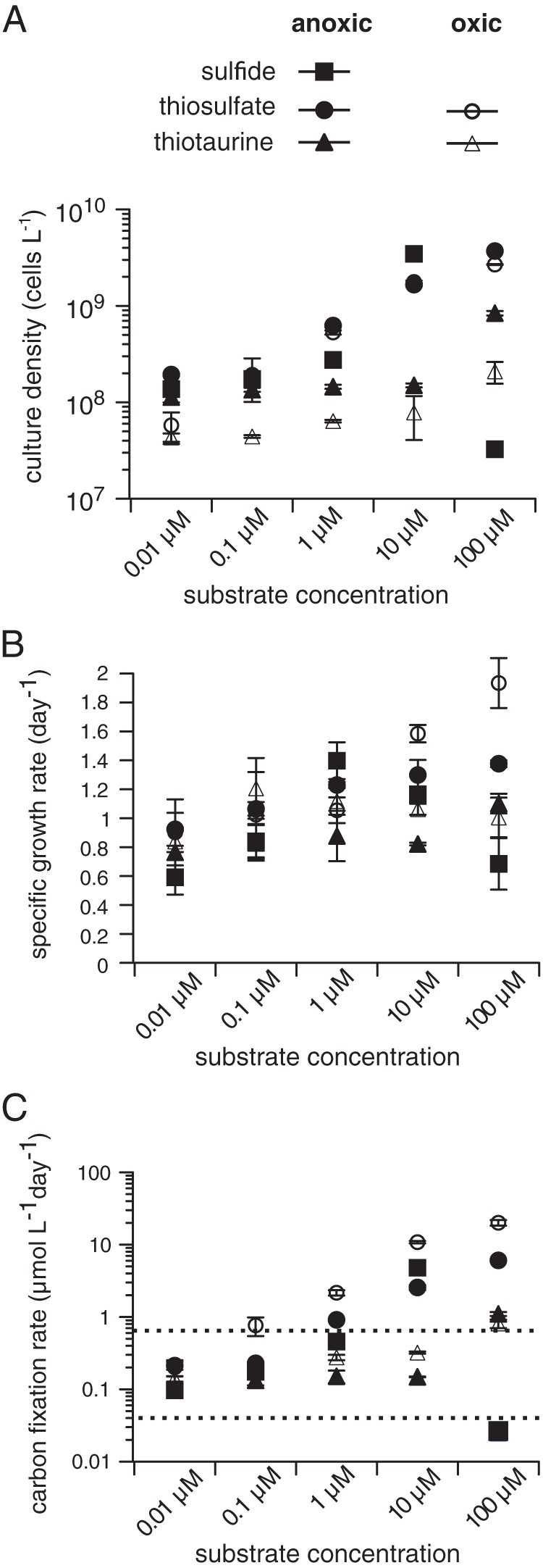
Specific growth and carbon fixation rates for “*Ca*. Thioglobus autotrophicus” under various growth conditions. (A to C) Maximum culture cell density (A), specific growth rates (B) and carbon fixation rates (C) in media amended with 0.01, 1, 10, and 100 μM sulfide, thiosulfate, or thiotaurine. Each data point was calculated from individual growth curves conducted in triplicate (see also Fig. S2). Error bars (standard deviation [SD]) are either shown for each data point or are masked by the symbol. The dashed line in panel C corresponds to estimates of SUP05 carbon fixation in field studies (10, 11). All cultures were inoculated with cells that were starved for sulfur after two successive transfers into sulfur deplete media as previously described (15).

To determine the limits of growth of “*Ca.* Thioglobus autotrophicus” with respect to sulfur and oxygen concentrations, we grew cultures on different inorganic and organic sources of sulfur that may be present at low concentrations under both oxic and anoxic conditions. Cultures grew over a broad range of sulfide (anoxic), thiosulfate (oxic and anoxic), and thiotaurine (oxic and anoxic) concentrations (0.01 to 100 μM) but were unable to use other sources of reduced sulfur that were tested, such as the amino acid methionine or the sulfonates taurine and dimethylsulfoniopropionate (DMSP), which are often present in the environments that SUP05 cells inhabit (Fig. 1; see also Fig. S1 and S2 and Table S1 in the supplemental material). Cultures reached their highest cell densities in anoxic seawater media but had the highest specific growth rates under oxic growth conditions in thiosulfate-replete media (Fig. 1A and B and Table S1). The highest specific growth rates measured under anoxic growth conditions were in media with 1 μM sulfide added, whereas sulfide concentrations of >1 μM inhibited growth. Recent evidence of high-affinity sulfide uptake by SUP05 in anoxic marine waters indicates that there is a cryptic marine sulfur cycle operating at below-detection (nanomolar) substrate concentrations (20). Here, we show that sources of reduced sulfur other than sulfide, including organic sulfur, could support a cryptic sulfur cycle in oxygenated seawater with concentrations as low as 10 nM.

10.1128/mBio.00216-19.1 FIG S1 Growth of “*Ca*. Thioglobus autotrophicus” cells on reduced sulfur substrates. Growth experiments with 10 μM inorganic (thiosulfate, sulfide, or polysulfide) or 10 μM organic (cysteine, methionine, taurine, thiotaurine, hypotaurine, or dimethylsulfoniopropionate [DMSP]) sulfur sources. Growth experiments were started using cultures in exponential-growth phase that were starved for sulfur in two successively transfers, as previously described (15). All growth experiments were conducted in triplicate with a starting concentration of 10^3^ cells ml^−1^ each. Download FIG S1, EPS file, 2.0 MB. Copyright © 2019 Shah et al. 2019 Shah et al. This content is distributed under the terms of the Creative Commons Attribution 4.0 International license.

10.1128/mBio.00216-19.2 FIG S2 Growth of “*Ca*. Thioglobus autotrophicus” at various concentrations of reduced organic and inorganic sulfur; relates to Fig. 1. (A to E) Growth curves on sulfide, thiosulfate, and thiotaurine under anoxic growth conditions (A to C) and on thiosulfate and thiotaurine under oxic growth conditions (D and E). Growth experiments were started using cultures that were in exponential-growth phase and that were starved for sulfur after two successive transfers into sulfur-depletes media, as previously described (15). All growth experiments were conducted in biological triplicate with a starting concentration of 10^3^ cells ml^−1^ each. Error bars (SD) are either shown for each data point or are masked by the symbol. Download FIG S2, EPS file, 2.0 MB. Copyright © 2019 Shah et al. 2019 Shah et al. This content is distributed under the terms of the Creative Commons Attribution 4.0 International license.

10.1128/mBio.00216-19.7 TABLE S1 Growth characteristics of “*Ca*. Thioglobus autotrophicus.” Cells were enumerated over a broad range of reduced sulfur substrate concentrations (0.01 to 100 μM). Sulfide, thiosulfate, and thiotaurine were electron donors; nitrate was the terminal electron acceptor under anoxic growth conditions, and thiosulfate and thiotaurine were electron donors under oxic growth conditions. Download Table S1, DOCX file, 0.1 MB. Copyright © 2019 Shah et al. 2019 Shah et al. This content is distributed under the terms of the Creative Commons Attribution 4.0 International license.

We calculated carbon fixation rates for “*Ca*. Thioglobus autotrophicus” using culture cell densities, growth rates, and measurements of cellular carbon content (Tables 1 and S1) to compare “*Ca*. Thioglobus autotrophicus” carbon fixation rates with estimates of SUP05 carbon fixation in the field. Estimates of carbon fixation in marine OMZs suggest that SUP05 populations fix between 0.013 and 0.592 μmol C liter^−1 ^day^−1^ (10) in anoxic waters. Estimates for “*Ca*. Thioglobus autotrophicus” carbon fixation ranged from 0.03 (±0.01) to 6.06 (±0.10) μmol C liter^−1 ^day^−1^ in anoxic seawater media and from 0.14 (±0.01) to 20.1 (±1.79) μmol C liter^−1 ^day^−1^ in oxic seawater media (Fig. 1C and Table S1). However, at substrate concentrations more typical of sea water (≤1 μM), “*Ca*. Thioglobus autotrophicus” carbon fixation rates ranged from 0.1 (±0.02) to 0.91 (±0.02) μmol C liter^−1 ^day^−1^ in anoxic seawater media and 0.14 (±0.01) and 2.16 (±0.22) μmol C liter^−1 ^day^−1^ in oxic seawater media. Evidence that submicromolar concentrations of reduced organic and inorganic sulfur sources fuel chemoautotrophic carbon fixation at relatively high rates and across oxic-anoxic boundaries significantly increases the dark carbon fixation potential of SUP05 in the oceans.

**TABLE 1:** Summary of “*Ca*. Thioglobus autotrophicus” strain EF1 cell size and sulfur storage measurements and CHN analyses^*a*^

“*Ca*. Thioglobus autotrophicus” characteristic	Data by condition	
	Anoxic (nitrate)	Oxic (oxygen)
Cell diam (μm)^*b*^	0.462 ± 0.03	—^*c*^
Cell vol (μm^3^)	0.056 ± 0.01	0.22 ± 0.22
Cytoplasmic vol (μm^3^)	0.039 ± 0.01	0.1 ± 0.11
Sulfur globule diam (μm)	0.156 ± 0.01	0.36 ± 0.16
Sulfur globule vol (μm^3^)	0.002 ± 0.001	0.031 ± 0.002
Carbon content/cell (fg)^*d*^	9.92 ± 0.63	32.2 ± 1.21
Nitrogen content /cell (fg)	2.29 ± 0.22	9.33 ± 0.47
Hydrogen content /cell (fg)	0.11 ± 0.01	1.67 ± 0.03

a All growth experiments were conducted in triplicate using filter-sterilized seawater media amended with thiosulfate as the electron donor and with nitrate or oxygen as the electron acceptor.

b Cryo-EM measurements.

c —, polymorphic cells.

d CHN analyses (unit fg = 10^−15^ g).

### Adaptive morphology and physiology.

**FIG 2:**
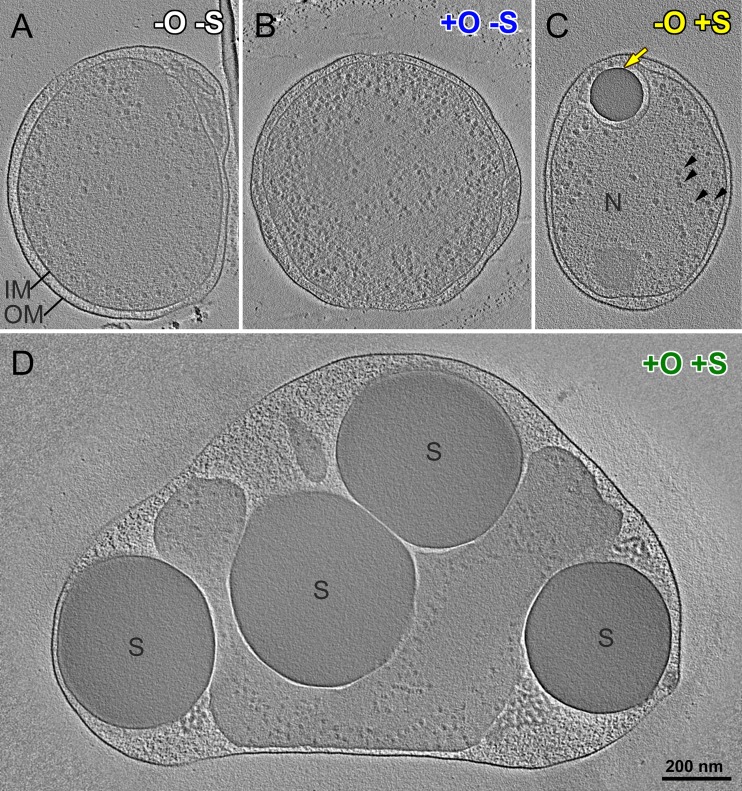
“*Ca*. Thioglobus autotrophicus” cell morphologies under various growth conditions. (A to D) Cryo-electron tomographic slices of three-dimensional (3D)-reconstructed cells grown under anoxic (−O) (A) and oxic (+O) (B) growth conditions in sulfur-depleted (−S) media and under anoxic (C) and oxic (D) growth conditions in sulfur-replete (+S) media. Note that all cells are displayed at the same scale (scale bar in panel D = 200 nm). Black arrowheads, ribosome-sized particles; yellow arrow, electron-dense sulfur globule (S) in the periplasmic space, i.e., between their inner and outer membrane (IM and OM, respectively); N, the nucleoid.

**FIG 3:**
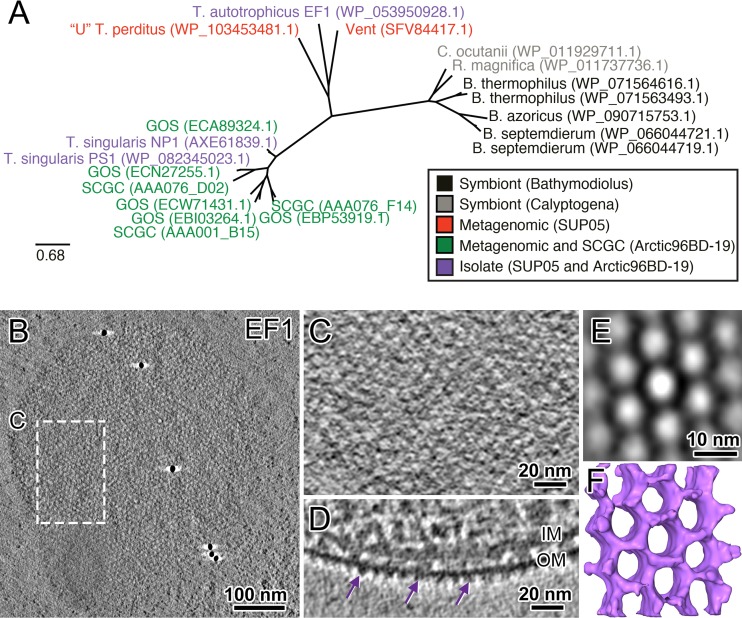
Monomolecular S-layers in SUP05 strains. (A) Maximum likelihood tree constructed from S-layer protein sequences using the RAxML (49) model GTRGAMMA to find the best tree topology (100 replicates) (see also Fig. S6 for other strains). Sequences in the tree are color coded according to their source: symbionts, metagenomes, single-cell amplified genomes (SCGC), or isolates. NCBI protein accession numbers are in parentheses. (B) Cryo-electron tomographic slice of an intact rapidly frozen and 3D-reconstructed “*Ca*. Thioglobus autotrophicus” strain EF1 cell, showing the symmetric pattern of the S-layer. (C) Zoom-in of the S-layer pattern outlined in panel B. (D) A tomographic side view of the S-layer shows the proteins protruding on the outer side of the outer cell membrane (OM). (E and F) A tomographic slice (E) and isosurface rendering (F) of the subtomograph average of the S-layer clearly shows its hexagonal pattern, with a pore diameter of ∼10 nm.

We used cryo-ET and label-free quantitative proteomics to explore responses at the cellular and molecular levels (Fig. 2 and 3 and Movie S1). We observed dramatic shifts in phenotype, including shifts in morphology and protein expression of “*Ca*. Thioglobus autotrophicus” EF1 cells under the different growth conditions. Cryo-ET images showed dramatic changes in cell size, shape, and molecular structures when cells are grown under different conditions (Fig. 2, S3, and S4 and Table 1). Cell volumes increased on average by 390%, from 0.056 to 0.22 μm^3^, when EF1 cells used thiosulfate as an energy source under oxic growth conditions (Fig. 2D and S3G and H) compared to anoxic growth conditions (Fig. 2C and S3A and B and Table 1). The enlarged cells contained numerous large and relatively electron dense globules in the periplasmic space (Fig. 2D, S3G and H, and S4D to F). A similar single and smaller globule was observed in cells grown under anoxic conditions with a sulfur source (Fig. 2C, S3A and B, and S4A to C). However, cells grown without a sulfur source under oxic or anoxic conditions lacked these globules (Fig. 2A and B and S3C to F).

10.1128/mBio.00216-19.9 MOVIE S1 3D-reconstructed and graphically modeled “*Ca*. Thioglobus autotrophicus” cell. The movie shows a sequence of cryo-tomographic slices through a 3D-reconstructed “*Ca*. Thioglobus autotrophicus” cell that was grown on natural seawater media and under anoxic growth conditions, i.e., with nitrate as the terminal electron acceptor, and with a sulfur source present. Color coding of the graphical model is as follows: blue, outer membrane; green, inner membrane; red, cytoplasm with ribosome-sized particles (white); yellow, sulfur globule. Download Movie S1, MOV file, 3.0 MB. Copyright © 2019 Shah et al. 2019 Shah et al. This content is distributed under the terms of the Creative Commons Attribution 4.0 International license.

10.1128/mBio.00216-19.3 FIG S3 Two additional examples of “*Ca*. Thioglobus autotrophicus” EF1 cells that were 3D reconstructed by cryo-ET are shown for the four different growth conditions; relates to Fig. 1. (A to H) Tomographic slices of 3D-reconstructed “*Ca*. Thioglobus autotrophicus” cells cultured under anoxic conditions (−O) with a sulfur source present (+S) (A and B), anoxic conditions without a sulfur source in the media (−S) (C and D), oxic conditions without a sulfur source (+O −S) (E and F), and oxic conditions with a sulfur source (+O +S) (G and H). All cells are displayed at the same scale (scale bar in panel H = 200 nm); cells grown with a sulfur source present store electron-dense sulfur globule(s) (yellow arrows) in their periplasmic space, i.e., between their inner and outer membrane (IM and OM, respectively). N, nucleoid; arrowheads, ribosome-sized particles. It is not clear if the electron-dense particle in panel D is a nascent/remnant of a sulfur granule (light-yellow arrow). Download FIG S3, JPG file, 2.5 MB. Copyright © 2019 Shah et al. 2019 Shah et al. This content is distributed under the terms of the Creative Commons Attribution 4.0 International license.

10.1128/mBio.00216-19.4 FIG S4 Typical size range of sulfur globules found in “*Ca*. Thioglobus autotrophicus” cells. (A to F) Tomographic slices displayed at the same magnification (scale bar in panel F = 100 nm) show the 3D-reconstructed sulfur globules (yellow arrows) in “*Ca*. Thioglobus autotrophicus” cells cultured with a sulfur source present in the media. Note that under anoxic conditions (−O) (A to C), the sulfur globules (S) were smaller than those under anoxic conditions (+O) (D to F). IM, inner membrane; OM, outer membrane. Download FIG S4, JPG file, 2.4 MB. Copyright © 2019 Shah et al. 2019 Shah et al. This content is distributed under the terms of the Creative Commons Attribution 4.0 International license.

10.1128/mBio.00216-19.6 FIG S6 The S-layer structures in “*Candidatus* Thioglobus singularis” strains visualized by cryo-ET. (A to E) S-layer in “*Ca.* Thioglobus singularis” PS1. (A) A tomographic slice of an intact frozen-hydrated “*Ca.* Thioglobus singularis” PS1 cell shows the S-layer pattern on the cell surface. (B) Zoom-in of the outlined S-layer in panel A. (C) Tomographic side view of the S-layer and outer membrane (OM). (D and E) A tomographic slice (D) and isosurface rendering (E) of the subtomogram average of the S-layer clearly shows its hexagonal pattern, with a pore diameter of ∼5 nm. (F to J) S-layer in “*Ca.* Thioglobus singularis” NP1. (F) A tomographic slices of an intact frozen-hydrated “*Ca.* Thioglobus singularis” NP1 cell shows the S-layer pattern on the cell surface. (G) Zoom-in of the outlined S-layer in panel F. (H) Tomographic side view of the S-layer (purple arrows) on the outside of the outer membrane (OM). (I and J) A tomographic slice (I) and isosurface rendering (J) of the subtomogram average of the S-layer clearly shows its hexagonal pattern, with a pore diameter of ∼10 nm. IM, inner membrane. Download FIG S6, JPG file, 2.6 MB. Copyright © 2019 Shah et al. 2019 Shah et al. This content is distributed under the terms of the Creative Commons Attribution 4.0 International license.

Sulfur-oxidizing bacteria that store elemental sulfur have paralogous genes that encode sulfur globule membrane proteins and also genes for the production and consumption of the sulfur that is stored in globules. We identified four putative genes for sulfur globule proteins (s*gp*) in the “*Ca*. Thioglobus autotrophicus” genome (Fig. S5), as well as genes required to use stored sulfur (*dsrEFH* and *dsrC*) (21). The overall size and combined volume of periplasmic sulfur globules exhibited a 15.5-fold increase during thiosulfate-replete oxic growth, from 0.002 to 0.031 μm^3^. Under anoxic and oxic thiosulfate-depleted growth conditions, cells grew to the same small size as under the anoxic thiosulfate-replete condition but stored little to no sulfur (Fig. 2A and B and S3C to F). Morphological changes associated with the accumulation of large sulfur storage globules can fuel growth in times of sulfur starvation, increasing fitness across redox gradients.

10.1128/mBio.00216-19.5 FIG S5 Sulfur globule proteins (Sgp) in SUP05. A maximum likelihood tree was constructed from putative sulfur globule protein sequences using the RAxML (49) model GTRGAMMA to find the best tree topology (100 replicates). Sequences in the tree are color coded according to their source: symbionts, metagenomes, single-cell amplified genomes (SCGC), or isolates. NCBI protein accession numbers are in parentheses. Download FIG S5, EPS file, 1.3 MB. Copyright © 2019 Shah et al. 2019 Shah et al. This content is distributed under the terms of the Creative Commons Attribution 4.0 International license.

### Conserved monomolecular surface layer proteins in SUP05.

Specialized cell structures are necessary to accommodate such vastly different morphotypes, particularly with respect to the cell envelope and cell division. We identified a putative surface layer protein (S-layer)-coding gene in the “*Ca*. Thioglobus autotrophicus” genome (19) and verified homologous sequences in other SUP05 species (free-living and symbiont) (Fig. 3A). Sequences in a maximum likelihood phylogenetic tree grouped according to known differences in 16S rRNA genes (14). We then used cryo-electron tomographs of “*Ca*. Thioglobus autotrophicus” cells and related SUP05 species to confirm that there is a paracrystalline protein arrangement with hexagonal symmetries associated with the outer surface of SUP05 cell membranes (Fig. 3B to F and S6). These images show that all known cultures of the genus “*Candidatus* Thioglobus” (strains EF1, PS1, and NP1) form a protein S-layer around the cells. Interestingly, the pore sizes of the hexagonal S-layer pattern are not conserved within the subclades and range from 5 to 10 nm in diameter (Fig. 3D and F and S6D, E, I, and J).

**FIG 4:**
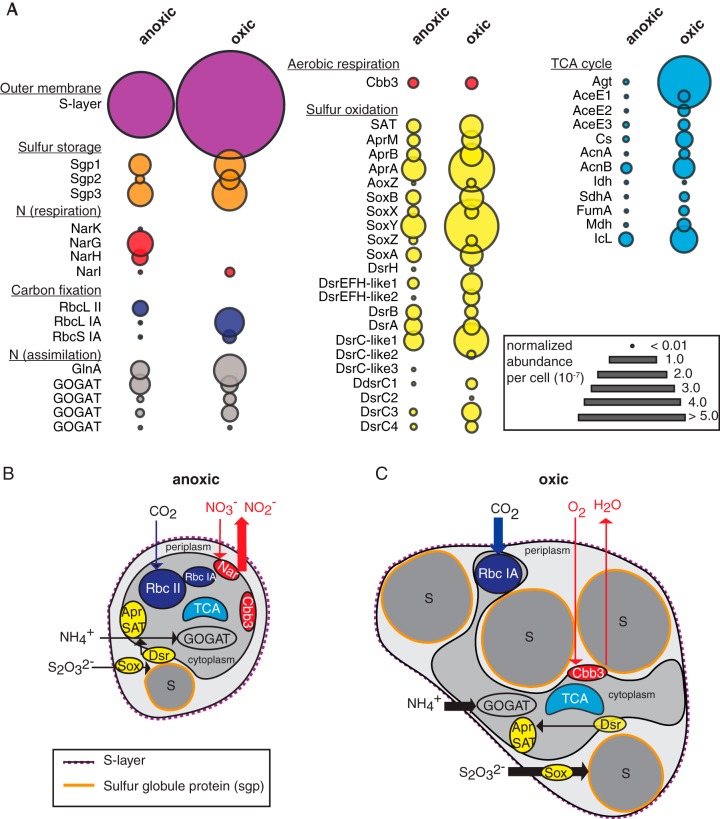
Protein expression in “*Ca*. Thioglobus autotrophicus” cells under anoxic and oxic growth conditions in thiosulfate-replete media. (A) Normalized relative protein abundance per cell of the S-layer protein and other key proteins involved in carbon, nitrogen, and sulfur metabolisms under anoxic and oxic growth conditions. (B and C) Schematic of “*Ca*. Thioglobus autotrophicus” cell structure and metabolism under anoxic (B) and oxic (C) growth conditions. Schematics are based on cell size, CHN content, and protein expression-level data. Major processes are color coded as in panel A for carbon, nitrogen, and sulfur metabolic pathways. Arrows indicate direction and magnitude of major nutrient fluxes. Sgp, sulfur globule protein; Nar, dissimilatory nitrate reductase; Rbc, ribulose-1,5-bisphosphate carboxylase/oxygenase; Gln, glutamine synthetase; GOGAT, glutamine oxoglutarate aminotransferase; Cbb3, cytochrome *c* oxidase (Cox); SAT, ATP sulfurylase; Apr, adenosine phosphosulfate reductase; Sox, sulfur oxidation system; Dsr, dissimilatory sulfite reductase system. Additional information about the identified proteins is available in Dataset S1.

Consistent with cryo-electron tomography data, proteomic analysis revealed that the putative 34-kDa S-layer protein was the most highly expressed protein under both anoxic and oxic growth conditions and was more highly expressed in the enlarged morphology (Fig. 4A, outer membrane S-layer). S-layers in bacteria and archaea have important roles in maintaining cell shape and integrity during cell division, in regulating host-symbiont interactions, and in initiating mineral aggregation, as a zone for exoenzyme adhesion, and as a mechanism for motility (22–25). S-layer gene and protein sequences are highly varied and often species specific, making them difficult to verify from genomic data alone. We used a combination of genomic, visualization, and proteomics data to demonstrate that S-layers are conserved in cultured representatives from the SUP05 clade. Our analyses suggest that these uncharacterized components of the SUP05 outer cell wall are conserved and likely essential to maintain the structural integrity of the cell.

### FtsZ-less cell division in a free-living proteobacterium.

The large accumulations of sulfur require morphological changes that could complicate other cellular functions, such as cell division. Interestingly, we found that “*Ca*. Thioglobus autotrophicus” strain EF1 does not code for the structural protein FtsZ, a tubulin homolog that is essential for cell division in all known free-living proteobacteria (26). The only known proteobacteria that lack FtsZ are uncultured endosymbionts, including members of the SUP05 clade (27–29). Earlier evidence of genome reduction in SUP05 endosymbionts led to the suggestion that they have eliminated FtsZ and other structural proteins that are required to deal with osmotic stress in more stable host cell environments (27). Upon closer inspection of the SUP05 genomes, we found that they lack known structural proteins that regulate cell division in other organisms (FtsZ, Cdv, actin, and dynamin). We also found that SUP05 cultures code for and express the rod-determining protein MreB (Dataset S1), which has an essential role in regulating cell division in the obligate intracellular and FtsZ-less Chlamydia pathogens (30). Thus, the SUP05 division appears to be more similar to other FtsZ-less bacteria in the *Planctomycetes*-*Verrucomicrobia*-*Chlamydiae* (PVC) superphylum than to other free-living proteobacteria, as previously suggested for SUP05 symbionts (29). This suggests that the S-layer likely has roles in maintaining cell structure and in regulating cell division, which has been demonstrated in lobed archaea (31). If so, this represents a novel form of cell division in free-living proteobacteria.

10.1128/mBio.00216-19.8 DATASET S1 Raw and normalized protein identifications for “*Ca*. Thioglobus autotrophicus” grown under anoxic and oxic growth conditions. Metadata for all protein identifications, including but not limited to protein descriptions, protein length, subcellular localization, COG category, HMM protein description, peptides identified, % protein coverage, protein probabilities, spectral counts, and normalized abundance/cell. Download Data S1, XLSX file, 0.5 MB. Copyright © 2019 Shah et al. 2019 Shah et al. This content is distributed under the terms of the Creative Commons Attribution 4.0 International license.

### Differential protein expression under oxic and anoxic growth conditions.

Based on the observed growth and morphological differences, we also expected that protein expression levels of key biochemical pathways would differ between the oxic and anoxic growth conditions (Fig. 4A and Dataset S1). Of the 802 proteins detected, 81 (10%) were expressed only under anoxic growth conditions, and 135 proteins (17%) were expressed only under oxic growth conditions. The majority of identified proteins shared across growth treatments had higher relative protein abundance per cell under oxic growth (96%), suggesting that the larger cells are not only storing more sulfur but that they are also more biochemically active. Increases in expression under oxic growth were observed for proteins involved in central carbon, nitrogen, and sulfur metabolism (Fig. 4). Proteins required for aerobic respiration (Cbb3) or associated with carbon fixation in oxygenated waters (RbcL IA) were more highly expressed under oxic growth conditions. In contrast, essential proteins for anaerobic nitrate respiration (Nar) and anaerobic carbon fixation (RbcL II) were only detected under anoxic growth conditions. We also verified the differential expression levels of three proteins associated with sulfur storage globules (Sgp) and of proteins associated with the production and consumption of stored sulfur (Sox and Dsr) (Fig. 4) (21). This molecular evidence, together with visual evidence that the periplasmic globules are depleted when cells are starved for sulfur, indicates that the globules store elemental sulfur that can be used as an energy source when more reduced forms of sulfur are depleted. Our protein expression data confirm that EF1 uses nitrate as the terminal electron acceptor during anoxic growth and that cells use oxygen as the terminal electron acceptor, oxidize stored sulfur, and express an oxygen-tolerant variant of the RuBisCO protein under oxic growth conditions. Respiration using oxygen as the terminal electron acceptor conserves more energy than nitrate. It is likely that this extra energy associated with aerobic respiration is used to support sulfur storage. Reduced forms of sulfur, such as sulfide, are oxidized abiotically in the presence of oxygen. This ability to store reduced sulfur as a chemoautotrophic energy source extends the range of SUP05 dark carbon fixation to the oxygenated ocean.

### Implications of alternative cell sizes and morphologies in OMZs.

Callbeck and colleagues found that SUP05 cells with the genetic potential to respire oxygen were active when they were transported offshore, providing a partial explanation for cryptic sulfur cycling in OMZs (10). Using estimates of cell size, they calculated the volumes and carbon content of SUP05 cells in Peru upwelling waters. Their volume estimates ranged from 0.18 ± 0.01 μm^3^ in onshore OMZ waters to 0.39 ± 0.04 μm^3^ in offshore eddy-influenced OMZ waters. Their carbon estimates ranged from 61 to 83 fg carbon/cell in the same waters, respectively. To compare our results with these field estimates, we quantified the carbon, nitrogen, and hydrogen contents of “*Ca*. Thioglobus autotrophicus” cells. We found that the increases observed in “*Ca*. Thioglobus autotrophicus” cell size under sulfur-replete oxic growth conditions were not only due to increases in sulfur storage globules but were also due to increases in biomass. We found that oxic-grown “*Ca*. Thioglobus autotrophicus” cells contained 3 times more carbon (9.92 versus 32.2 fg C/cell), 5 times more nitrogen (2.29 versus 9.33 fg N/cell), and 14 times more hydrogen (0.11 versus 1.67 fg H/cell) than anoxic-grown cells, respectively (Table 1). This suggests that the larger SUP05 cells identified in offshore waters that were reported by Callbeck and colleagues (10) may have been respiring both oxygen and nitrate under low-dissolved-oxygen (microaerophilic) conditions. Although their estimates of cellular carbon content are likely high, their data suggest that SUP05 cells assimilate more biomass when oxygen is available for growth. Our data indicate that cells store more sulfur and assimilate more biomass when oxygen is available for aerobic respiration.

Morphology has been used as a trait to classify bacteria since they were first seen and described by Antonie Van Leeuwenhoek in the late 1600s. Although newer structural, functional, and evolutionary approaches are now used to classify bacteria and their relationships, morphology remains an important selectable trait that can affect the survival and environmental impacts of microorganisms (32, 33). Cell size across bacterial taxa can vary by several orders of magnitude, from the ultrasmall bacterium “*Candidatus* Pelagibacter ubique,” which is adapted to ocean environments where nutrients are often limiting (34–36), to the giant bacterium Thiomargarita namibiensis that concentrates nitrate in a large central vacuole to sustain growth in marine sediments (37–39). However, the potential for size and morphology to significantly alter elemental cycling within bacterial taxa has been largely overlooked. Here, we show that sulfur stored in intracellular globules alters the morphology and metabolism of a facultative aerobic SUP05 isolate, enabling cells to persist when environmental sulfur is not available. This highlights the adaptive roles of SUP05, with significant implications for ocean C, N, and S cycling (Fig. 4B and C).

## MATERIALS AND METHODS

### Anoxic and oxic growth conditions.

Cells were grown as previously described (15, 19, 40), with minor modifications. Media was prepared by filter sterilizing seawater collected from the Puget Sound using a tangential flow filtration system equipped with a 30-kDa filter (Millipore, NJ, USA). Media was stored, checked for purity, and subsequently distributed into acid-washed and autoclaved 125-ml glass serum bottles. Oxic media bottles were covered with sterilized aluminum foil. Anoxic media bottles were sealed with 20-mm butyl rubber stoppers (Wheaton, Millville, NJ, USA) and bubbled with an N_2_-CO_2_ gas mixture that contained 1,000 ppm CO_2_ (Praxair, Danbury, CT, USA) for 10 min, and then the headspace was sparged for an additional 5 min. Oxygen removal was verified by adding GasPak anaerobic strips (BD, Franklin Lakes, NJ, USA) to uninoculated serum bottles. Batch cultures used for cryo-electron tomography and CHN analysis were amended with additional 5 μM NH_4_^+^ and 30 μM NO_3_^−^ to ensure the highest cell yields.

### Thiosulfate-depleted growth conditions.

Thiosulfate-depleted cells were required to evaluate growth with alternative electron donors. This was achieved by successively transferring cells into natural unamended seawater media, as previously described (15). Briefly, cells from cultures in exponential-growth phase were transferred from natural seawater media amended with 1 mM thiosulfate to unamended seawater media (1st transfer). The cultures were incubated for 4 days, at which time the cells were transferred to fresh unamended seawater media (2nd transfer) and incubated for 4 days. Cells from transfer 2 (T2) were deemed thiosulfate depleted. This was verified under oxic and anoxic growth conditions by conducting a thiosulfate depletion experiment in triplicate and by adding a third transfer (3rd). No growth was detected after three transfers under anoxic conditions. Incubations were at 13°C.

### Reduced-sulfur growth experiments.

Growth on different sulfur compounds was determined under anoxic and oxic growth conditions by adding thiosulfate-depleted cells to serum bottles containing 10 μM reduced sulfur (thiosulfate, sulfide, polysulfide, cysteine, methionine, taurine, thiotaurine, hypotaurine, or dimethylsulfoniopropionate [DMSP]) (Fisher Bioreagents, WA, USA; Wako Chemicals, USA). DMSP was obtained from the Moran research group at the University of Georgia. Growth with different concentrations of reduced sulfur was determined under anoxic growth conditions by adding thiosulfate-depleted cells to serum bottles containing 0.01, 0.1, 1.0, 10, or 100 μM reduced sulfur (thiosulfate, sulfide, and thiotaurine). Only anoxic conditions were tested for sulfide, since it oxidizes in the presence of oxygen. All growth experiments were conducted in biological triplicates incubated at 13°C and had the same initial cell concentration (1 × 10^3^ cells ml^−1^).

### Cryo-electron tomography.

Frozen-hydrated specimens were prepared as described previously (34). Briefly, “*Ca*. Thioglobus autotrophicus” strain EF1 cells were harvested by centrifuging at 39,000 × *g* at 4°C for 1 h and then resuspended in 100 μl seawater media. Quantifoil grids (copper, R2/2) with holey carbon film and lacey carbon grids were positively glow discharged for 30 s at 30 mA. Three-microliter volumes of cell solution were added onto the grid, and then the cells were mixed with 1 μl of a 10-fold concentrated solution of 10-nM bovine serum albumin (BSA)-colloidal gold (Sigma-Aldrich, St. Louis, MO) (41). Excess fluid was blotted from the back side of the grid with Whatman filter paper for ∼2 s, before plunge-freezing the grid in liquid ethane at about −170°C using a homemade plunge-freezing device.

Cells were imaged using a Titan Krios 300-kV field emission gun (FEG) transmission electron microscope (FEI Company, Hillsboro, OR) equipped with a postcolumn imaging filter (Gatan, Pleasanton, CA) and Volta phase plate (FEI). Images were recorded at ×26,000 magnification using a K2 Summit direct electron detector (Gatan) with an effective pixel size of 5.5 Å. Tilt series were acquired using the Serial EM microscope control software (42) under low-dose mode while tilting the sample −60° to +60° in 2° increments. The cumulative electron dose was ∼100 e^−^/Å. The defocus was set to 0 μm while using the phase plate to increase image contrast. The energy filter was in zero-loss mode with a slit width of 20 eV. The tilt series images were aligned using the IMOD software package using the 10-nm gold particles as fiducial markers (43). We used the IMOD package for the tomographic reconstructions by weighted back-projection and for graphic modeling and the UCSF Chimera software package for isosurface rendering of the average S-layer (44).

### Carbon, hydrogen, and nitrogen measurements.

Oxic and anoxic cultures were filtered at the highest cell yield (early stationary phase) on 25-mm (baked at 450°C for 4.5 h) GFS75 glass fiber filters (Fisher Scientific, NJ, USA). Due to the varied pore sizes of glass fiber filters, the loss of cells during filtration was quantified by measuring cell concentration in the media pre- and postfiltration. Filtration of oxic cultures resulted in a total of 6.50 × 10^9^ cells collected on the filter (4.5% lost in the filtrate), whereas filtration of anoxic cultures resulted in 1.46 × 10^10^ cells collected on the filter (11% lost in the filtrate). Uninoculated media was also filtered to prepare controls that were used to account for background carbon, hydrogen, and nitrogen contamination. Filters were stored inside aluminum foil (baked at 450°C for 4.5 h) and frozen at −80°C. CHN content in filters was analyzed using an Exeter CE-440 CHN autoanalyzer. Final values of carbon, nitrogen, and hydrogen per cell were adjusted using control values (less than 0.05%).

### Protein extraction and analysis by liquid chromatography and tandem mass spectrometry.

Cellular biomass from “*Ca*. Thioglobus autotrophicus” strain EF1 cultures grown under either oxic or anoxic conditions was collected onto filters (separately), and proteins were extracted from filters using bead-beating and freeze-thaw cycles to lyse cells. Control filters containing only the media (amended Puget Sound water) were processed as sample controls. Lysed cells were suspended in RapiGest SF (Waters) to help facilitate protein solubilization and then underwent disulfide reduction with Tris(2-carboxyethyl)phosphine, alkylation with iodoacetamide, and finally, in-solution protease digestion with a trypsin/Lys-C mix (Promega). Following established manufacturer protocols, RapiGest was hydrolyzed, and samples were desalted using a MacroSpin C_18_ column (NestGroup). Desalted samples were concentrated to near dryness using a centriVap concentrator, and all samples including controls were resuspended in 25 μl of a solution containing an internal standard of synthetic peptides (Waters Hi3 Escherichia coli Standard Waters; 50 fmol/μl). Samples were analyzed in triplicate by Waters ACQUITY M-Class ultrahigh pressure liquid chromatography (UPLC) coupled to a Thermo Q Exactive HF Orbitrap high-resolution mass spectrometer (HRMS) equipped with a nano-electrospray (NSI) source. All analyses were carried out in positive mode at an NSI spray voltage of 2.0 kV, and data were collected using data-dependent acquisition (DDA) on the top 10 ions. All LC-HRMS conditions were identical to those previously described by Qin et al. (45).

### Protein identification and label-free quantitation.

Data processing was conducted using the software from the Trans-Proteomic Pipeline (TPP version 5.1.0) (46). Briefly, database searching was performed using COMET (47, 48) with a FASTA protein database for “*Ca*. Thioglobus autotrophicus” strain EF1 (UniProt 9GAMM; 1,488 proteins accessed February 2017; 1,506 KEGG estimated protein coding genes), the E. coli chaperone protein (Hi3 standard; UniProt accession number P63284), and a concatenated set of randomized sequences. Additional COMET parameters included trypsin enzyme specificity, one allowed nontryptic termini, up to two missed cleavage sites, carbamidomethylation of cysteine residues as a fixed modification, and oxidation of methionine residues and clipping of N-terminal methionine residues as variable modifications. Files were then searched in PeptideProphet with greater than 90% peptide probability, followed by ProteinProphet and iProphet within the TPP. The final list of protein identifications was filtered to ensure that at least one peptide was observed consistently across individual injections and the protein probability within each replicate was greater than 95%. Data filtering based on these constraints was done using output from Abacus and further constrained by iProphet protein lists (Dataset S1). A total of 802 EF1 proteins were observed and used for comparative analyses, representing 53% of all the predicted proteins encoded in the EF1 genome and corresponding to 721 (48%) and 667 (44%) proteins under oxic and anoxic conditions, respectively.

Total spectral counts were averaged across triplicate injections, and the standard deviations of these averages were calculated for error for each condition. Of the 29 proteins detected in the media control, many had very high standard deviations, indicative of their presence as injection contaminants. A label-free quantitative comparison of relative protein abundances across EF1 growth treatments was facilitated by normalizing the averaged protein spectral counts both to the Hi3 internal standard (to account for differences in NSI ionization of peptides due to sample matrix affects) and to the amount of biomass injected on-column, here referred to as normalized protein abundance per cell.

### Data availability.

Proteomics data have been deposited to the ProteomeXchange Consortium via the PRIDE partner repository with the data set identifier PXD013243. Cell images and other data related to this project are archived online and available through The Biological and Chemical Oceanography Data Management Office (BCO-DMO). Cryo-ET data have been deposited to a public database (EMDataResource) with the following six accession codes: EMD 0507-0509 and 0511-0513.
