# Supplementary material for: Morphological Plasticity in a Sulfur-Oxidizing Marine Bacterium from the SUP05 Clade Enhances Dark Carbon Fixation
Source: mBio. 2019 May 7;10(3):e00216-19. doi: 10.1128/mBio.00216-19 (PMC6509183; doi:10.1128/mBio.00216-19)
Supplement: TABLE S1 [file mBio.00216-19-st001.docx]

**Table S1**

|  | **anoxic** | | | **oxic** | | |
| --- | --- | --- | --- | --- | --- | --- |
| **Concentration**  **(μM)** | **Specific growth**  **rate (d^-1^)** | **C fixation**  **(µmol L^-1^d^-1^)** | **Max**  **cell density**  **(L^-1^)** | **Specific**  **growth**  **rate (d^-1^)** | **C fixation**  **(µmol L^-1^d^-1^)** | **Max**  **cell density**  **(L^-1^)** |
| thiosulfate |  |  |  | thiosulfate |  |  |
| 0.01 | 0.92±0.11 | 0.21±0.03 | 1.94x10^8^±7.06x10^6^ | 0.90±0.23 | 0.20±0.05 | 5.77x10^7^±2.08x10^7^ |
| 0.1 | 1.06±0.05 | 0.23±0.01 | 1.81x10^8^±1.18x10^7^ | 1.02±0.29 | 0.77±0.22 | 1.93x10^8^±9.21x10^7^ |
| 1 | 1.23±0.02 | 0.91±0.02 | 6.25x10^8^±2.19x10^7^ | 1.05±0.09 | 2.16±0.18 | 5.30x10^8^±2.57x10^7^ |
| 10 | 1.30±0.10 | 2.56±0.21 | 1.66x10^9^±7.62x10^7^ | 1.58±0.06 | 10.8±0.41 | 1.77x10^9^±5.49x10^7^ |
| 100 | 1.38±0.02 | 6.06±0.10 | 3.70x10^9^±1.14x10^8^ | 1.93±0.17 | 20.1±1.79 | 2.68x10^9^±2.67x10^7^ |
| thiotaurine |  |  |  | thiotaurine |  |  |
| 0.01 | 0.76±0.17 | 0.10±0.02 | 1.13x10^8^±1.34x10^7^ | 0.84±0.07 | 0.14±0.01 | 4.32x10^7^±4.27x10^6^ |
| 0.1 | 0.83±0.12 | 0.13±0.02 | 1.35x10^8^±6.44x10^6^ | 1.21±0.21 | 0.21±0.04 | 4.42x10^7^±1.38x10^6^ |
| 1 | 0.88±0.17 | 0.15±0.03 | 1.45x10^8^±6.94x10^6^ | 1.12±0.09 | 0.28±0.02 | 6.38x10^7^±2.01x10^6^ |
| 10 | 0.82±0.01 | 0.15±0.00 | 1.52x10^8^±7.03x10^6^ | 1.06±0.04 | 0.32±0.01 | 7.83x10^7^±3.77x10^7^ |
| 100 | 1.10±0.07 | 1.10±0.07 | 8.40x10^8^±4.50x10^7^ | 1.01±0.14 | 0.81±0.11 | 2.09x10^8^±5.29x10^7^ |
| sulfide |  |  |  | sulfide *NA | | |
| 0.01 | 0.59±0.12 | 0.10±0.02 | 1.37x10^8^±8.37x10^6^ |  |  |  |
| 0.1 | 0.84±0.12 | 0.17±0.03 | 1.75x10^8^±1.28x10^7^ |  |  |  |
| 1 | 1.40±0.13 | 0.46±0.04 | 2.75x10^8^±1.08x10^7^ |  |  |  |
| 10 | 1.17±0.03 | 4.80±0.12 | 3.46x10^9^±1.70x10^8^ |  |  |  |
| 100 | 0.68±0.18 | 0.03±0.01 | 3.26x10^7^±4.98x10^6^ |  |  |  |

* No data for oxic growth on sulfide
