# Supplementary figures and images for: Morphological Plasticity in a Sulfur-Oxidizing Marine Bacterium from the SUP05 Clade Enhances Dark Carbon Fixation
Source: mBio. 2019 May 7;10(3):e00216-19. doi: 10.1128/mBio.00216-19 (PMC6509183; doi:10.1128/mBio.00216-19)

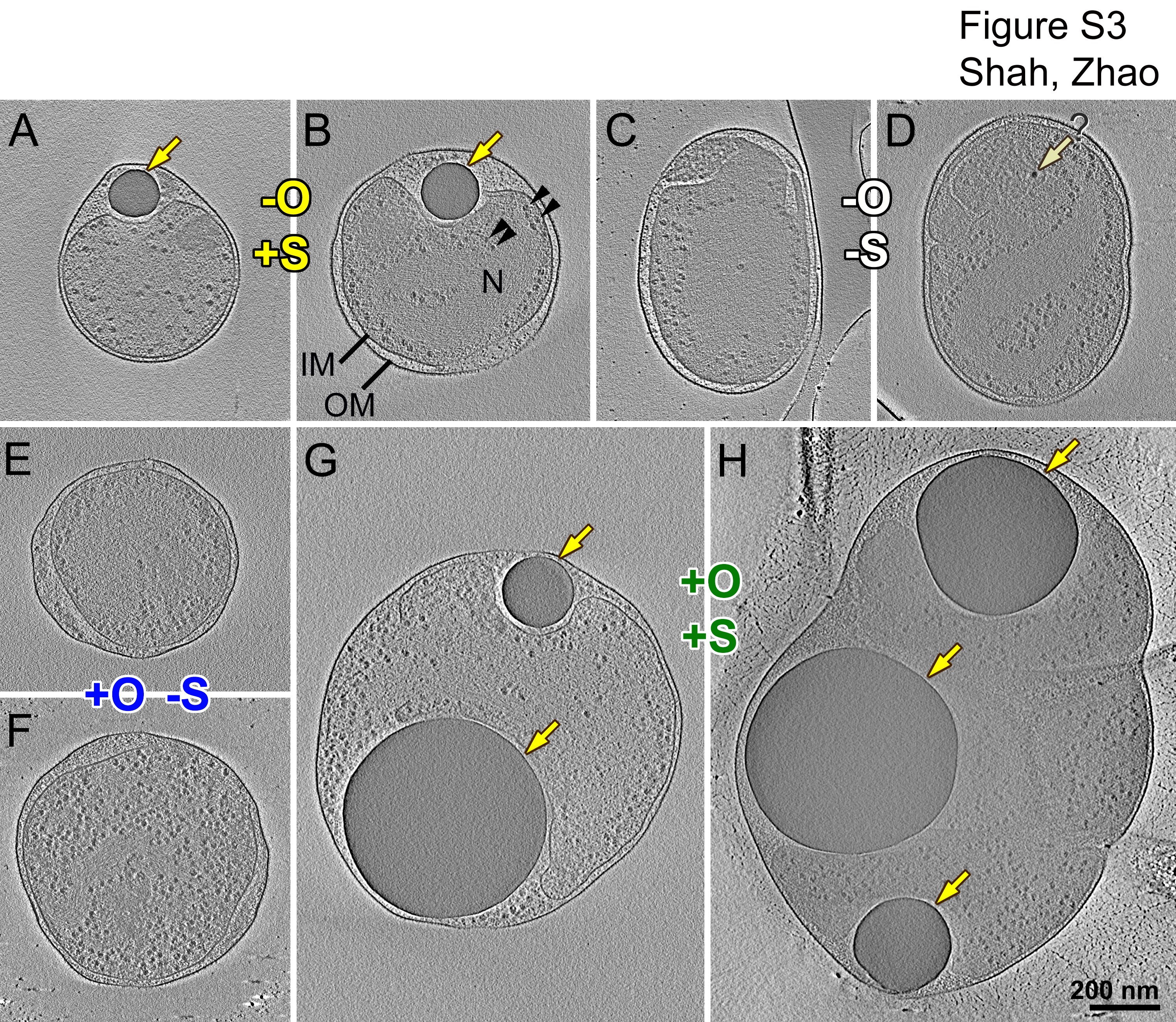

Supplement: FIG S3 [file mBio.00216-19-sf003.jpg]

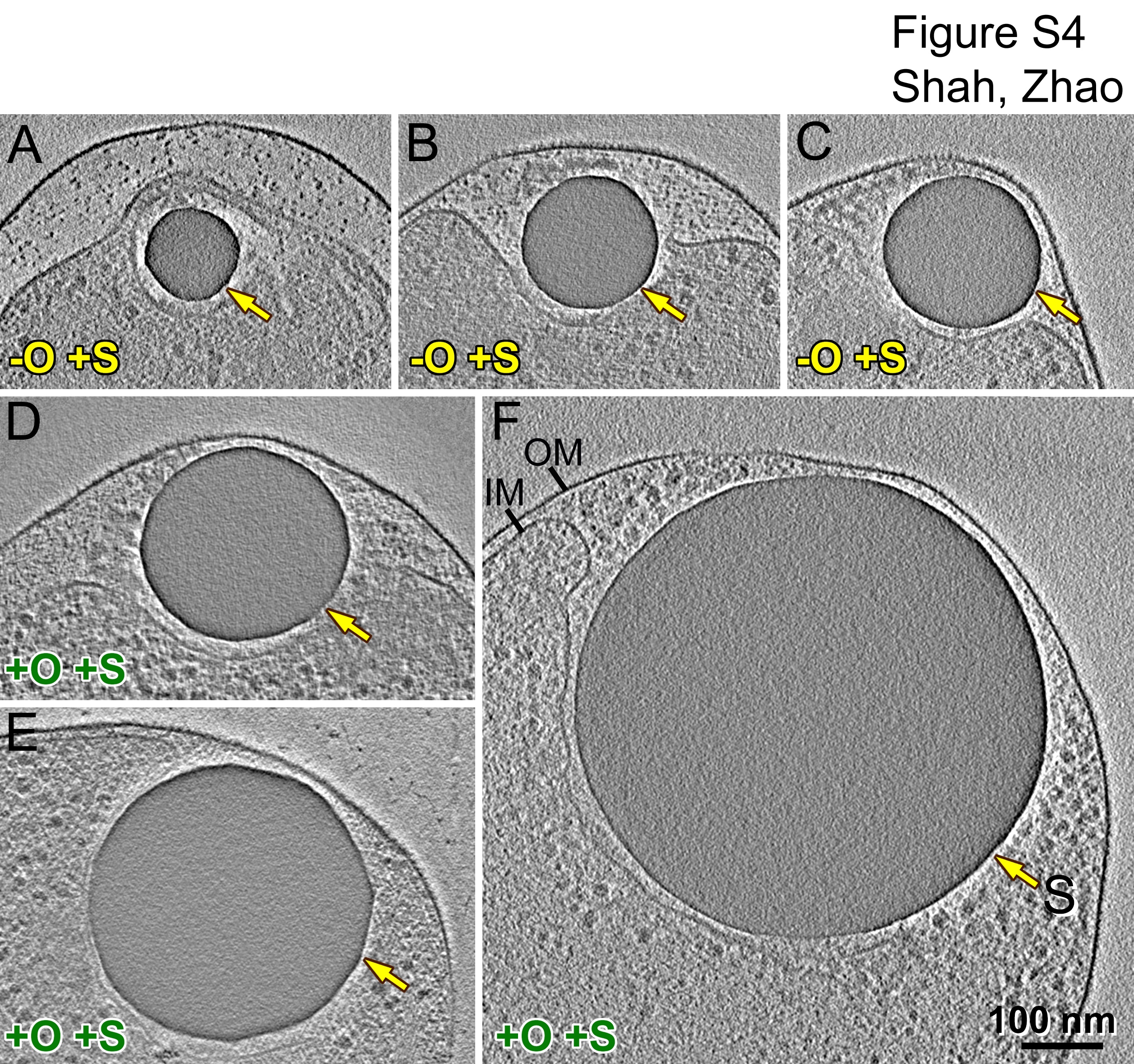

Supplement: FIG S4 [file mBio.00216-19-sf004.jpg]

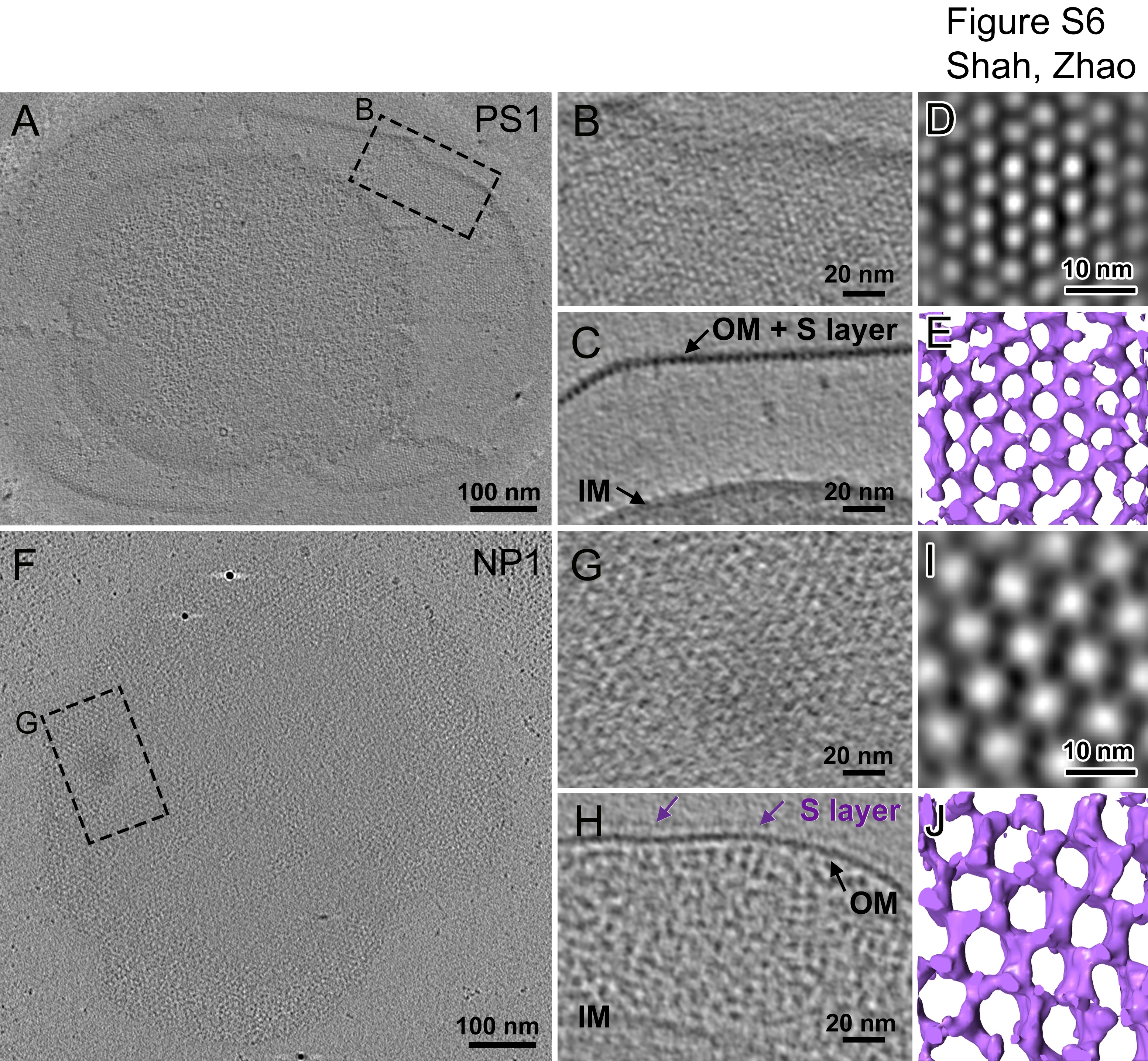

Supplement: FIG S6 [file mBio.00216-19-sf006.jpg]
